# Antibiofilm and antimetabolic effects of disinfectants on *Pseudomonas aeruginosa* strains isolated from cosmetic manufacturing environments

**DOI:** 10.1007/s00253-026-13719-y

**Published:** 2026-01-26

**Authors:** Kamila Korzekwa, Daria Kowalczyk-Chrząstowska, Aleksandra Szmurło, Maciej Wernecki, Agnieszka Ulatowska-Jarża, Igor Buzalewicz, Dorota Wojnicz

**Affiliations:** 1 Department of Microbiology, Faculty of Biological Sciences, University of Wroclaw, Wroclaw, Poland; 2 Department of Biomedical Engineering, Faculty of Fundamental Problems of Technology, Wroclaw University of Science and Technology, Wroclaw, Poland; 3 Department of Biology and Medical Parasitology, Faculty of Medicine, Wroclaw Medical University, Wroclaw, Poland

**Keywords:** Biofilm eradication, Cosmetics industry, Disinfectants, Hypochlorous acid, *Pseudomonas*, SLES

## Abstract

**Abstract:**

*Pseudomonas aeruginosa* is a major contaminant in cosmetics, posing significant health risks to consumers. These bacteria form biofilms that protect them from disinfectants commonly used in the cosmetic industry. This study aimed to assess the impact of disinfectants, cleaners, and sanitizers on the metabolic activity and biofilm formation of *P. aeruginosa* isolated from the production line in a cosmetic manufacturer. In vitro experiments were conducted using silicone, Teflon, ethylene propylene diene monomer and acid-resistant steel surfaces, which are commonly used materials in cosmetic production line equipment. Spectrophotometric methods were used to evaluate biofilm formation and metabolic activity, while different imaging techniques (SEM, EFM, OCT) were employed to visualize biofilm structure directly on examined surfaces. The results showed that hypochlorous acid is the most effective disinfectant in inhibiting biofilm formation. Hypochlorous acid significantly reduced the metabolic activity of *P. aeruginosa*, particularly in biofilms forming on the surface made of stainless steel. Additionally, the study validated the feasibility of implementing a sterilization method using hypochlorous acid directly in industrial conditions for production line sterilization. Results showed a significant reduction in contamination levels in water passing through the installation, from an uncountable level to below 0.1 × 10^1^ CFU mL^−1^. In conclusion, the effectiveness of disinfectants in preventing biofilm formation and metabolic activity is dependent on their composition and the type of surface on which the biofilms form. Hypochlorous acid proves to be an effective disinfectant for combating bacterial biofilms in the cosmetic industry.

**Key points:**

- *The tested strains of P. aeruginosa formed a strong biofilm and were drug-resistant*
- *Hypochlorous acid exhibited the highest antibacterial efficacy among the tested agents*
- *Hypochlorous acid effectively eliminated biofilm-associated microbes in the facility’s water system*

## Introduction

Microbiological control is a key element in ensuring consumer health safety and maintaining high product quality in the cosmetic industry. Cosmetic products, especially those containing water or organic ingredients, provide a favorable environment for microbial growth. The presence of microorganisms in cosmetics can lead to adverse effects such as skin irritation, infections, and in more severe cases, significant health risks. Routine microbiological testing enables early detection of pathogens, whose presence in cosmetic products poses a potential threat to end users. Microbiological contamination also affects the physicochemical stability of the product—it may cause changes in odor, color, and consistency, and reduce the effectiveness of active ingredients, thereby lowering the overall efficacy and usability of the cosmetic. Important sources of contamination include plant-based raw materials, water systems, and technological installations. Microorganisms that are not effectively removed during cleaning and disinfection processes can form biofilms—complex microbial structures with increased resistance to chemical agents—which pose a serious threat to product quality and consumer safety.

One of the most frequently identified bacteria in biofilms is *Pseudomonas aeruginosa*, an opportunistic pathogen capable of rapidly forming biofilms on technological surfaces. The removal of biofilms presents a significant technological challenge, as their structure considerably reduces the effectiveness of conventional disinfectants (Ludensky 2003). *P. aeruginosa* is a microorganism that can resist many antibiotic groups. The presence of antibiotic-resistant strains, as reported by Shaqra et al. (2014), exacerbates the epidemiological risk associated with their presence in consumer products (Shaqra et al. 2014).

Despite the use of advanced deionized water purification systems, such as ozonation, 0.2 µm microfiltration, and ultraviolet irradiation, microorganisms may survive within water systems and subsequently enter technological installations and the external environment. Results from our own studies confirmed the presence of antibiotic-resistant *P. aeruginosa* strains in production lines of a cosmetic manufacturing facility, underscoring the need for effective decontamination strategies.

The aim of the study was to evaluate the efficacy of various disinfecting, washing, and cleaning agents in limiting the development of biofilms and the metabolic activity of *P. aeruginosa* strains isolated from production lines. In particular, the study focused on the impact of hypochlorous acid on biofilm formation and its morphology on surfaces made of materials commonly used in cosmetic production equipment (silicone, Teflon, ethylene propylene diene monomers, and acid-resistant steel). The research was conducted both in vitro and directly on the production line of a cosmetic manufacturing plant, allowing for an assessment of the practical effectiveness of the disinfection methods under real-world conditions. Based on the results obtained from in vitro studies, the decision was made to use hypochlorous acid to flush the water system employed in the cosmetics production process at a cosmetics manufacturing facility, with the aim of disinfection and biofilm removal.

## Materials and methods

### Bacterial strains

All bacterial strains used in this study are listed in Table 1. The study used the reference strain *P. aeruginosa* PAO1, whose genome has been completely sequenced and which is most commonly used in the analysis of the genetics, physiology, and metabolism of *P. aeruginosa* (Chandler et al. 2019). The remaining *P. aeruginosa* strains were taken from a process plant, made from acid-resistant steel, transporting the raw material—Sodium Laureth Sulphate (SLES). Bacteria labelled with SLES1 (*P. aeruginosa* 448) were taken from elbows introducing the raw material into loops, and bacteria labelled with SLES2 (*P. aeruginosa* 304) were taken from the ends of such loops. The other two *P. aeruginosa* strains numbered 1999 and 2096 were isolated during standard culture of the final product, resulting from the testing schedule in place at the cosmetics manufacturing facility. Species identification of *P. aeruginosa* was confirmed using MALDI-TOF MS (Bruker, Germany).

**Table 1:** *Pseudomonas aeruginosa* strains used in this study

Strain species	Laboratory strain number	Sources
*Pseudomonas aeruginosa*	PAO1 (ATCC 15692)	----------
*Pseudomonas aeruginosa*	448	SLES 1
*Pseudomonas aeruginosa*	304	SLES 2
*Pseudomonas aeruginosa*	1999	Calendula extract
*Pseudomonas aeruginosa*	2096	Shower gel

### Bacterial DNA isolation

The bacterial cultures were incubated overnight at 28 °C in 3 mL of Luria broth (bioMérieux, France). Genomic DNA extraction from these cultures was performed using the Genomic Mini AX Bacteria Spin Kit. The integrity and concentration of the extracted DNA were assessed by UV–Vis spectrophotometry using a NanoPhotometer NP80 (Implen, Germany) and fluorometric analysis using a Qubit 2.0 fluorometer with the Qubit™ 1X dsDNA High Sensitivity Assay Kit (Invitrogen, Life Technologies, USA).

### Genome sequencing

Whole genome sequencing (WGS) of bacterial isolates was performed using nanopore sequencing technology (NST). Libraries were prepared using the Rapid Barcoding Kit 24 V14 (SQK-RBK114.24, Oxford Nanopore Technologies, UK). Sequencing was performed on a MinION Mk1B instrument using a Flongle Flow Cell R10.4.1 (FLO-FLG114, Oxford Nanopore Technologies, UK) together with the Flongle Sequencing Expansion Kit (EXP-FSE002, Oxford Nanopore Technologies, UK). Basecalling of the raw sequencing data was performed using Dorado 0.5.3 and the DNA model (dna_r10.4.1_e8.2_400 bps_sup@v4.3.0), with subsequent demultiplexing performed using the same tool. Read filtering was performed with Chopper (De Coster and Rademakers 2023) passing reads with a minimum Phred average quality score of 13 and a minimum length of 200 bp. Genome assembly was carried out using Flye 2.9.3—b1797 (Kolmogorov et al. 2019) and further polished using Medaka 1.11.3. Taxonomic analysis of the bacterial strains was carried out using the Type (Strain) Genome Server (TYGS, available at: https://tygs.dsmz.de) (Meier-Kolthoff and Göker 2019). Type strains closely related to four *Pseudomonas* strains were identified using the MASH algorithm (Ondov et al. 2016). Pairwise genome comparisons were conducted using Genome Blast Distance Phylogeny (GBDP) (Meier-Kolthoff et al. 2013), followed by phylogenetic inference via FASTME (Lefort et al. 2015) and species clustering on the basis of digital DNA‒DNA hybridization with d4 formula (dDDH d4) values from GGDC 4.0. The phylogenetic tree was visualized using FigTree v1.4.4. (available at: http://tree.bio.ed.ac.uk/software/figtree). Antimicrobial resistance genes were screened using ResFinder 4.5.0 (Bortolaia et al. 2020; Camacho et al. 2009) (database update: 2024-03−22, available at: http://genepi.food.dtu.dk/resfinder) and Resistance Gene Identifier (RGI 6.0.5) with Comprehensive Antibiotic Resistance Database (CARD 4.0.1) using Web portal (available at: https://card.mcmaster.ca) (Alcock et al. 2023). Prophage sequences in genomes were annotated using PHASTEST 3.0 (available at: https://phastest.ca) (Wishart et al. 2023). The raw sequencing reads and assembled genomes have been deposited in the European Nucleotide Archive as part of a study registered as PRJEB75548.

### Bacterial susceptibility to antibiotics

To assess the susceptibility of bacterial strains to ceftazidime (CAZ), gentamicin (GN), imipenem (IMP), and colistin (CL), the antimicrobial minimum inhibitory concentration (MIC) method was used by microdilution in MHB (EUCAST. The European Committee on Antimicrobial Susceptibility Testing 2024). The determined MIC value was the first antibiotic concentration where no growth was visually observed. The MIC values were compared with the interpretative standards for the cutoff values for assayed *P. aeruginosa* strains (EUCAST. The European Committee on Antimicrobial Susceptibility Testing 2024). The strains were classified into two MIC categories: susceptible and resistant.

### Preparation of bacterial suspensions

The bacteria were grown on tryptone soy agar (TSA) (bioMérieux, France) at 28 °C for 24 h. For each strain, a suspension with an optical density of 0.5 McFarland (approximately 1.5 × 10^8^ CFU mL^−1^) was prepared in Mueller–Hinton broth (MHB). Then, 100 μL of this suspension was added to 900 μL of MHB.

### Biofilm formation in the presence of disinfectants, cleaning and sanitizing agents

The disinfectants, cleaning and sanitizing agents used in this study are described in Table 2. The biofilm formation capacity was determined using the modified O’Toole and Kolter method (O’Toole 2011). The wells of the 96-well titration plates were filled with 180 μL of disinfectant, cleaning, or sanitizing agent at the concentrations recommended by the manufacturer (Table 2). Twenty microlitres of the prepared bacterial suspensions were added to the appropriate wells. Bacteria in the presence of disinfectant or cleaning or sanitizing agent were incubated at 28 °C for 24, 48, and 72 h. At the end of the incubation, bacteria not associated with the biofilm mass were removed from the wells by washing three times with a phosphate-buffered saline (PBS; bioMérieux, France). Then 125 μL of a 0.1% crystal violet solution was added to the wells and incubated for 15 min at 28 °C. In the next step, each well was rinsed again twice with 200 μL of PBS. Finally, 200 μL of 96% ethyl alcohol was added to the wells to dissolve the dye. OD measurements were made using the titration plate reader with a monochromator Asys Hitach UVM 340, Driver Version: 4.02 (ASYS HITECH GmbH, Austria) at a wavelength of 595 nm.

**Table 2:** Disinfectant, cleaning, and sanitizing agents used in this study

Type of agent	Concentration recommended by the manufacturer	Active ingredient	Contact time with the disinfected surface
Cleaner	2–5% (2%)	Sodium Alkylbenzenesulphonate, Tetrasodium Ethylene Diamine Tetraacetate	15 min
Sanitizer	1%	Hydrogen Peroxide, Acetic Acid, Peracetic Acid	30 min
Disinfectant 1	1%	N-alkyl Dimethyl Benzyl Ammonium Chloride	15 min
Disinfectant 2	3%	Hydrogen Peroxide	15 min
Disinfectant 3	100 ppm	Hypochlorous Acid	5 h

The sample containing bacterial suspension in MHB not treated with disinfectants, cleaning agents, and sanitizing agents was used as the positive control. The culture medium MHB without bacterial inoculum and disinfectant, cleaning agent, and sanitizing agent was used as a negative control.

On the basis of the OD value, bacterial strains were classified into one of the following groups: OD ≤ ODc—not producing biofilm; ODc < OD ≤ 2 × ODc—weak biofilm-producing; 2 × ODc < OD ≤ 4 × ODc—producing moderate biofilm; 4 × ODc < OD—producing strong biofilm. The ODc cutoff value was calculated as the sum of the mean OD value for the blank (MHB) and 3 times the standard deviation of the mean OD for MHB (Stepanović et al. 2004). The ODc value was 0.140.

### Efficacy of disinfectant, cleaning and sanitizing agents on the metabolic activity of bacteria living in preformed biofilms

To evaluate the effects of disinfectants and cleaning and sanitizing agents on the metabolic activity of the tested *P. aeruginosa* rods living in preformed biofilms, a 0.01% solution of 2,3,5-triphenyltetrazolium chloride (TTC; Merck, Germany) was used. Bacteria reduce TTC to red-colored formazan. The intensity of the red color increases with the amount of substrate used (Brown et al. 2013).

Twenty microliters of bacterial suspensions were added to the wells of a 96-well microtiter plate containing 180 μL of MHB. The plates were incubated for 24, 48, and 72 h at 28 °C to allow bacteria to form a biofilm. After these times, bacteria not associated with the biofilm mass were removed from the wells by washing three times with PBS. Then, 200 μL of MHB with disinfectant or cleaning or sanitizing agent at the concentrations recommended by the manufacturer (Table 2) was added to each well, and the mixture was incubated for 24 h at 28 °C. The control samples contained 200 μL of MHB. After incubation, the medium was discarded from each well, and the cells were thoroughly washed twice with PBS to remove unbound cells. A volume of 100 μL of 0.01% TTC was added to each well and incubated for 24 h at 28 °C. After incubation, the TTC solution was removed, and the microplate was air-dried. Bound red formazan was dissolved in 100 μL of a mixture of acetic acid and isopropanol at a ratio of 3:1. After 15 min of shaking (400 rpm) at room temperature, the amount of formazan produced by the bacteria was measured spectrophotometrically (Asys Hitach UVM 340, Driver Version: 4.02) at a wavelength of 490 nm. The reduction in bacterial metabolic activity was calculated according to the following formula:

$$Metabolic\;activity\;reduction\;(\%)\:=\:100\%\:-\:(value\;of\;the\;test\;sample\;absorbance/value\;of\;control\;sample\;absorbance)\:\times\:100\%$$

### Assessment of the impact of disinfectants, cleaning, and sanitizing agents on the metabolic activity of biofilm-associated bacteria on silicone, Teflon, EPDM and acid-resistant steel surfaces

Only bacterial strains isolated from SLES1 and SLES2 (*P. aeruginosa* strains 448 and 304) were used in this study because the cosmetics manufacturing plant from which these strains originate is struggling with bacterial biofilm development in pipelines transporting SLES. Due to the fact that, in the case of industrial cosmetics production, bacterial biofilms form directly on the surfaces of the components of the production line, it was decided to carry out tests on fragments of the gaskets (4 mm × 4 mm, surface area = 32 mm^2^) made of silicone, Teflon, ethylene propylene diene monomer (EPDM), and elements made of acid-resistant (stainless) steel installed in production line devices in the cosmetics industry.

Sterilized fragments of the gaskets and acid-resistant steel were placed in the wells of a 6-well plate containing MHB such that their part was above the surface of the medium. Then, 200 µL of bacterial suspension with a density of 0.5 McFarland was added to the wells. The mixture was incubated for 5 days at 28 °C. Bacterial suspensions were fed with MHB every 24 h. The tested materials were subsequently transferred to MHB medium containing appropriate concentrations of disinfectant, cleaning, and sanitizing agents recommended by the manufacturer (Table 2). The culture medium MHB without disinfectant, cleaning agent, and sanitizing agent was used as a control. After the specified incubation time at 28 °C (Table 2), the samples were transferred to a 0.1% TTC solution. After 4 h, the gaskets and acid-resistant steel fragments were again transferred to a solution containing isopropanol and acetic acid (3:1) to elute the red formazan formed after TTC reduction. The color intensity was read on a reader (Asys Hitach UVM 340, Driver Version: 4.02) at a wavelength of 490 nm.

### Visualization of bacterial biofilms using SEM, EFM, and OCT

To assess the structure and biomass of the biofilm formed directly on the surface of the examined materials, SEM (scanning electron microscopy), EFM (epifluorescence microscopy), and OCT (optical coherence tomography) were used. These imaging techniques were employed to qualitatively characterize the morphology of bacterial biofilms formed on the examined surfaces of silicone, Teflon, EPDM, and acid-resistant (stainless) steel. The SEM images show the microstructure of the biofilms, the EFM images show the extent to which the biofilm covers the surface under investigation, and the OCT tomograms show the permanent attachment of bacterial structures to the surface, indicating that we are studying the bacterial biofilms. Based on the obtained results, this examination was performed for disinfectant exhibiting the most antibacterial properties. In each case, the two kinds of samples (control and treated with hypochlorous acid) were examined. In the case of treated samples, the gaskets and acid-resistant steel with five-day-old biofilms were transferred to MHB medium containing hypochlorous acid at 100 ppm. After 5 h, the gaskets and acid-resistant steel were placed in a Petri dish and rinsed with PBS to remove hypochlorous acid.

#### SEM

Visualization of biofilm nano-/microstructures was performed using field emission scanning electron microscopy (Zeiss LEO 1550, Carl Zeiss NTS GmbH, Germany), and images were obtained using SmartSEM software (Carl Zeiss Microscopy, LLC, Germany). Five-day-old biofilms present on the gasket surfaces and acid-resistant steel were fixed with 2.5% (w/v) glutaraldehyde in 0.05 M sodium cacodylate buffer at 4 °C for 2 h. The samples were then washed in cacodylate buffer 3 times, 5 times each minute each time, and subsequently fixed with 1% (w/v) osmium tetroxide in cacodylate buffer for 1 h. Fixed samples were rinsed with cacodylate, buffered three times, and then dehydrated using gradient solutions of 25, 50, 70, 95, and 100% (v/v) ethanol for 10 min each, followed by critical spot drying with carbon dioxide. Tips were attached to the dried surfaces of SEM and covered with evaporated carbon. A voltage ranging from 1 to 5 kV was applied depending on the sample.

#### EFM

Microscopic examination was performed using an OPTIKA B-383FL microscope (OPTIKA S.r.l., Italy) equipped with a green excitation filter (510–550 nm), a dichroic mirror with a cutoff at 570 nm, and an emission filter of 575 LP. The objective magnification was set to 5×. Images were taken using an Opta-Tech MI5 FL camera (OPTA-TECH, Poland). The gaskets and acid-resistant steel with 5-day-old biofilms were placed in a Petri dish and rinsed with PBS to remove planktonic cells. A few drops of a 0.1% aqueous solution of Rose Bengal were then applied to completely cover the surface of the samples and stain the biofilm. After 5 min, the excess staining solution was removed, and the samples were washed three times with PBS to ensure the removal of any unbound dye.

#### OCT

In order to non-destructively assess the biofilm structures directly on the surfaces where they were formed, tomographic imaging was performed using spectral-domain optical coherence tomography (SD-OCT; Ganymede GAN611, Thorlabs, Lübeck, Germany) with a central wavelength of 930 nm and a high-resolution scanning lens kit (OCT-LK2-BB; objective: LSM02-BB; lateral resolution: 4 µm; field of view: 6 × 6 mm; working distance: 7.5 mm; Thorlabs, Lübeck, Germany). The axial resolution of the SD-OCT system was 4.2 µm in water, with an imaging depth of 2.2 mm in water. Tomogram registration and postprocessing of the obtained 3D volumetric data were performed using ThorImage OCT software (version 5.5.8.0, Thorlabs, Lübeck, Germany). Samples of 5-day-old bacterial biofilms on the surface of the test materials (silicone, Teflon, EPDM, and acid-resistant steel) were washed with PBS (phosphate-buffered saline) to remove planktonic cells. They were then placed in a PBS solution in a sterile µ-Dish (µ-Dish 35 low, IBIDI GmbH, Gräfelfing, Germany) and examined by SD-OCT. The A-scan rate of the SD-OCT system was 100 kHz. Each A-scan was averaged from 12 intermediate A-scans.

### Use of hypochlorous acid as a disinfectant in the water system of the production line

**Fig. 1:**
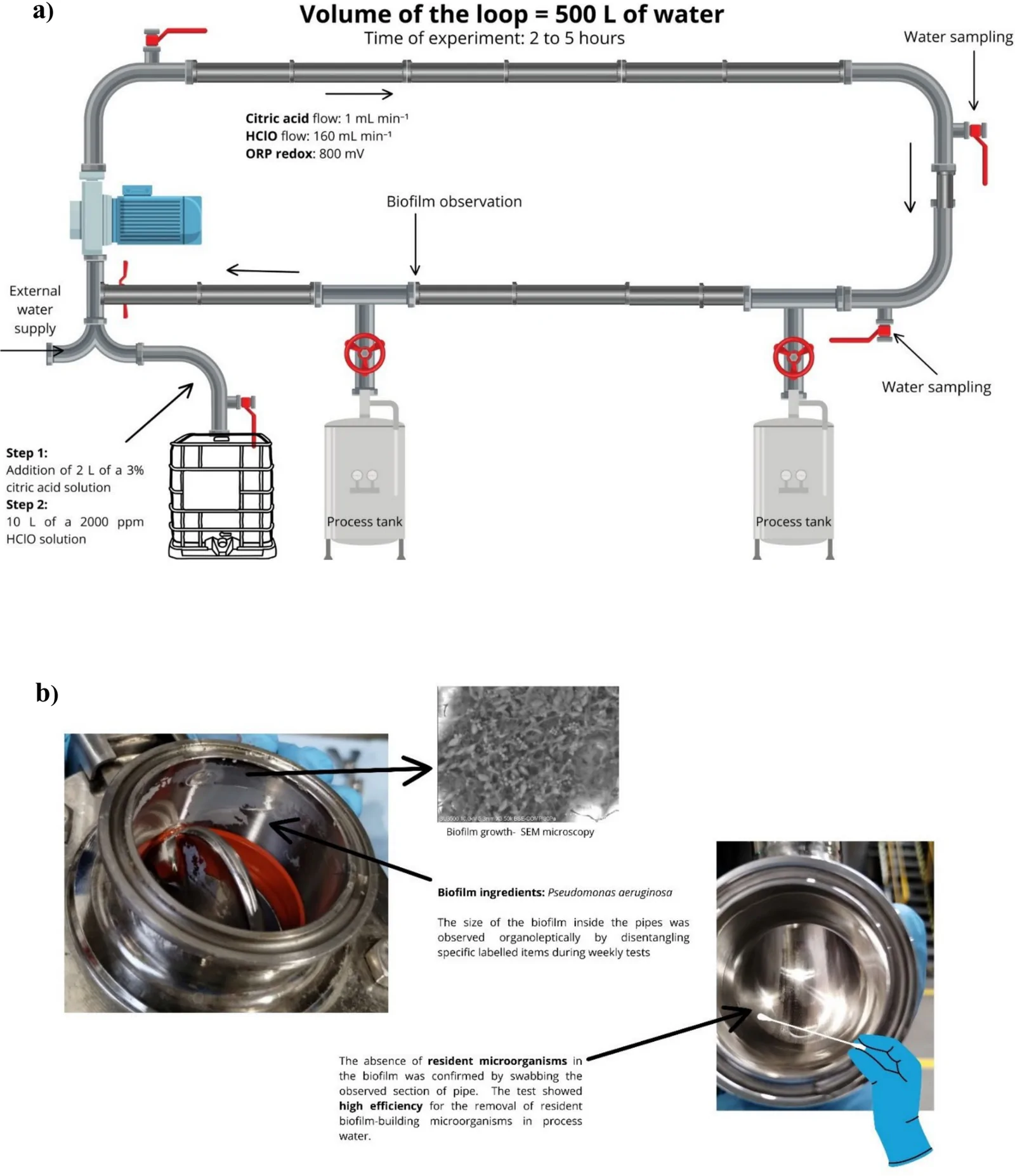
Facilities of the experiment (**a**). Taking swabs from biofilm formation sites in the water system (**b**)

To minimize the risk of biofilm formation on production line components and prevent potential contamination of cosmetic products, research was conducted on the practical use of hypochlorous acid (HClO) as an effective sterilization method. HClO solution was obtained by electrolysis of salt at a concentration of 2000 ppm. Tests were conducted on a technical scale after evaluating the antibiofilm effectiveness of HClO on bacterial biofilm grown in vitro. The entire water supply system, including the filtration system installed at the plant, was included in the study. The process was carried out over a period of 6 months, with the procedures repeated weekly on Mondays. The first step involved measuring the pH of the water entering the plant and acidifying it to pH 6 using citric acid. A 3% citric acid solution (2 L) was introduced into a 500 L water loop via a dosing pump. The dosing rate was set at 1 mL per minute to ensure uniform acid distribution throughout the system. After pH adjustment, samples were taken from all inlets in the loop to verify that the pH value was consistent across the system. Next, 10 L of 2000 ppm HClO solution was pumped into the water system at a flow rate of 160 mL min⁻^1^. After dosing, the solution circulated in the system for 15 min. Following this, the oxidation–reduction potential (ORP) was measured and maintained at 800 mV. The solution remained in the water system for a period of 2 to 5 h, with the duration gradually increased on subsequent days to assess the improvement in disinfection efficacy and the stability of the ORP parameter in the water. The experimental setup is shown in Fig. 1a. Each week, from Tuesday to Friday, samples were collected using a sterile swab from the gaskets of the water system, where bacteria were accumulating (Fig. 1b). The bacteria were suspended in 1 mL of PBS, followed by serial dilutions, which were plated onto nutrient agar plates. After 24 h, CFU per mL were counted.

### Statistical analysis

The nonparametric Mann–Whitney *U* test was used to analyze the obtained results. Statistical calculations were performed using Statistica 13.3 (Stat Soft, Krakow, Poland). All values are expressed as the means ± SD. The experiments were repeated three times. Values of *p* ≤ 0.05 were considered statistically significant.

## Results

### Genome sequencing and analysis

Whole-genome sequencing using nanopore sequencing produced complete, closed genome assemblies for *P. aeruginosa* strains 304 and 448 isolated from SLES (ENA accession numbers SAMEA115577706 and SAMEA115577705, respectively). While the genomes of strains 1999 and 2096 could not be assembled into complete circular chromosomes, the draft assembly of strain 2096 was sufficient for genome-based taxonomic identification.

**Fig. 2:**
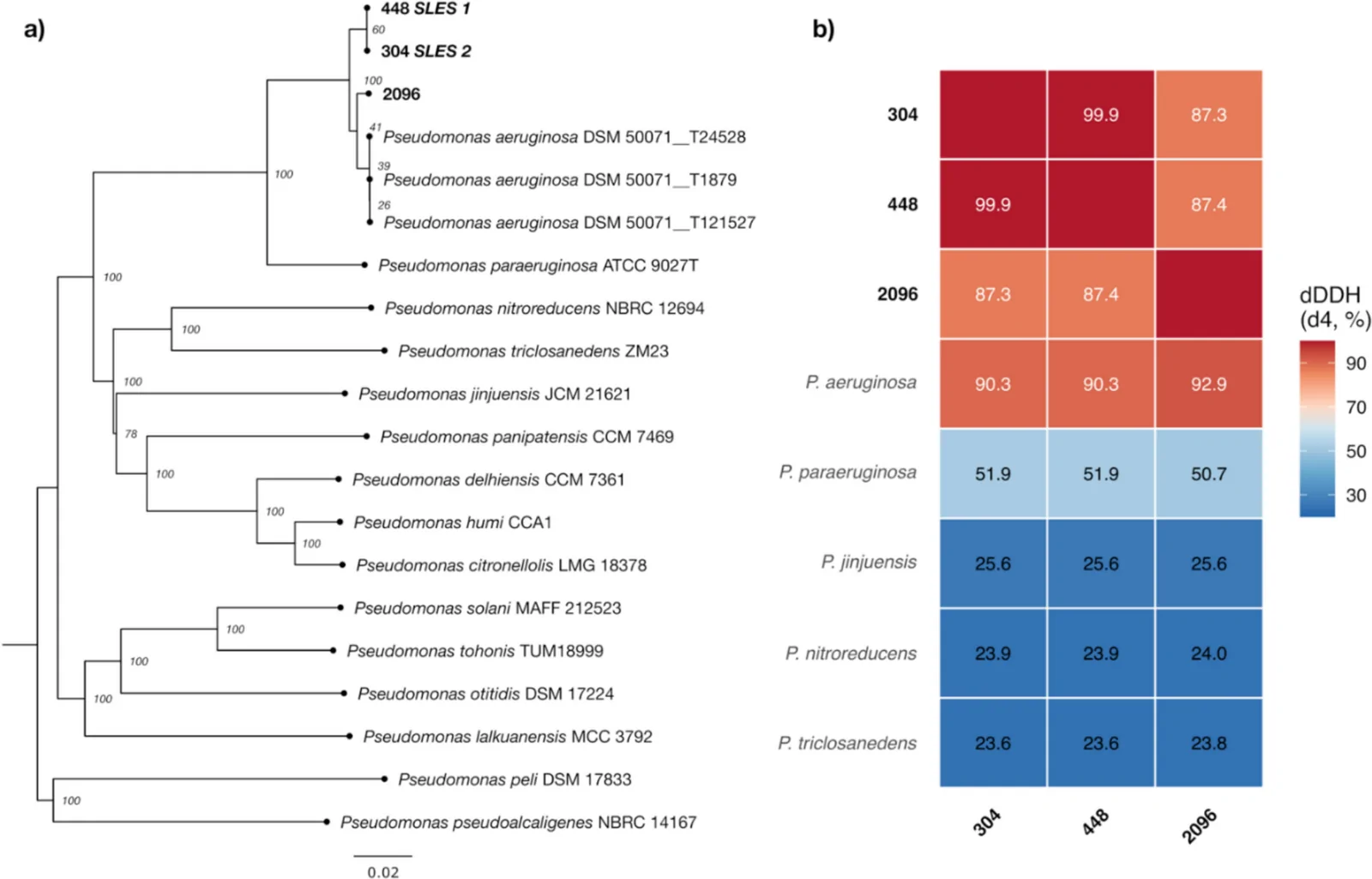
Phylogenomic analysis of *P. aeruginosa* strains isolated from cosmetic production facilities. **a** Genome BLAST Distance Phylogeny (GBDP) tree inferred using FASTME based on whole-genome sequences. The tree includes strains 304 (SLES2), 448 (SLES1), and 2096 isolated in this study (bold), along with *P. aeruginosa* DSM 50071 (SAMN03328803) type strain genome assemblies and related *Pseudomonas* species type strains. Bootstrap support values (%) based on 100 pseudo-bootstrap replicates are shown at nodes. Scale bar indicates 0.02 substitutions per site. **b** Heatmap of pairwise digital DNA-DNA hybridization (dDDH d4) values calculated using GGDC 4.0. Values above 70% indicate species-level similarity. The three study strains (304, 448, 2096) show >90% similarity to *P. aeruginosa* DSM 50071, confirming species identification. Strains 304 and 448 are nearly identical (99.9% dDDH)

Phylogenomic analysis using the Type (Strain) Genome Server (TYGS) revealed that strains 304 and 448 were nearly identical, with a digital DNA-DNA hybridization (dDDH d4) value of 99.9% (Fig. 2a, b). Both strains, together with strain 2096, grouped within the *P. aeruginosa* clade and showed dDDH d4 values above 90% when compared with the *P. aeruginosa* type strain DSM 50071 (90.3% for strain 304, 90.4% for strain 448, and 92.9% for strain 2096), confirming their species identification.

Despite their high overall similarity, some differences were identified between strains 304 and 448. Strain 448 contained a 48.6 kb prophage sequence showing the highest similarity to PHAGE_Pseudo_D3_NC_002484(37), inserted between the *trnR* and *folD* genes without disrupting the open reading frames. This prophage was not present in strain 304.

**Fig. 3:**
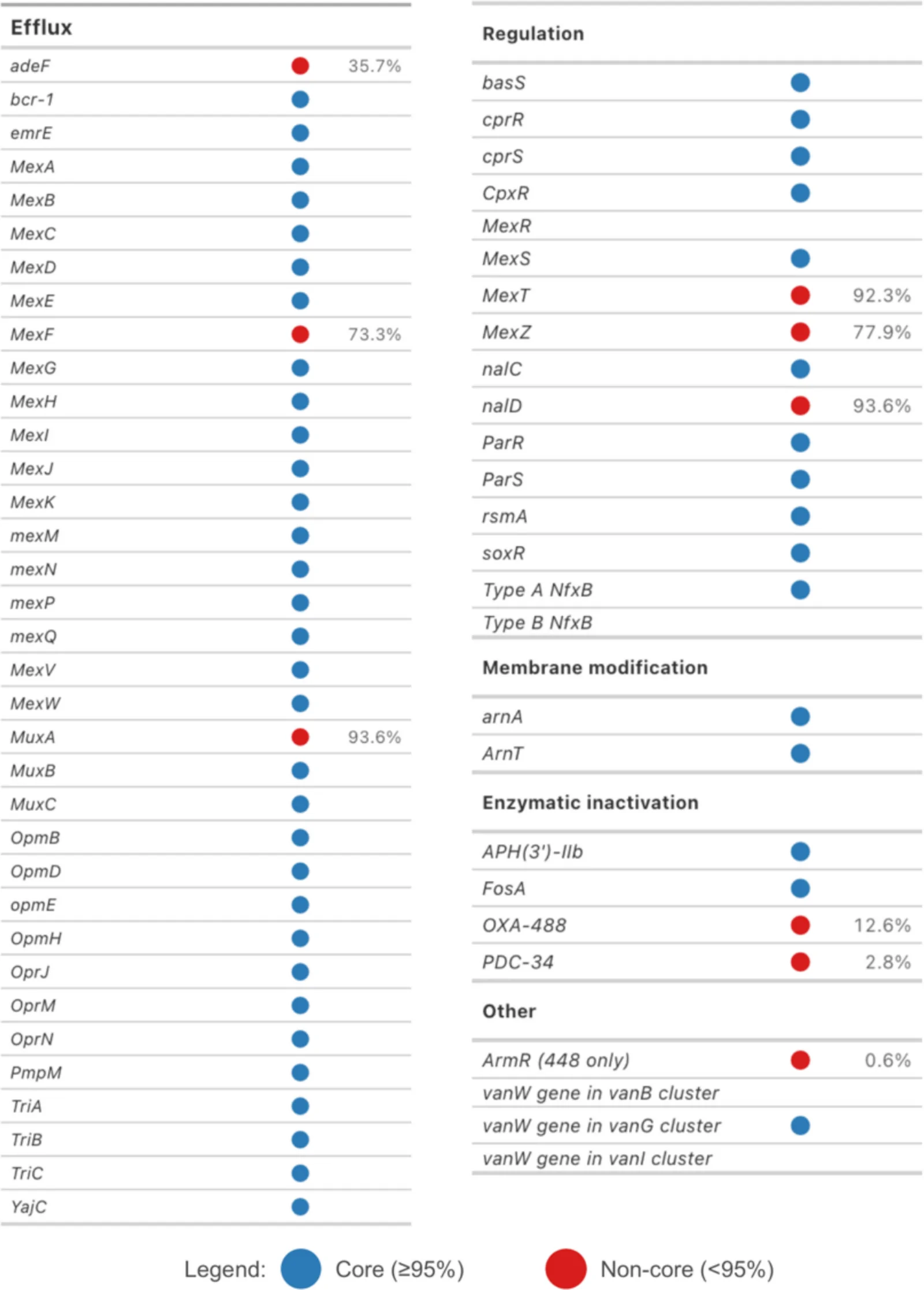
Antimicrobial resistance-associated genes detected in strains 304 and 448, annotated with general prevalence data for *P. aeruginosa* whole-genome sequences deposited in NCBI, as determined using the Comprehensive Antibiotic Resistance Database (CARD). Genes are grouped by functional category: efflux pump components, regulatory genes, membrane modification enzymes, enzymatic inactivation mechanisms, and other resistance determinants. Blue circles indicate core genes (prevalence > 95% in *P. aeruginosa* genomes), while red circles indicate non-core genes with lower prevalence, which may be responsible for less common phenotypes. The majority of resistance genes detected in strains 304/448 belong to the *P. aeruginosa* core genome. *ArmR* (regulator of MexXY-OprM) was detected only in strain 448

The Comprehensive Antibiotic Resistance Database (CARD) was used to determine whether the genomes contained any unusual or less common resistance-associated genes, as well as to provide data on the prevalence of these genes in *P. aeruginosa* genomes (Fig. 3). Most of the detected genes were classified as core *P. aeruginosa* genes with prevalence above 95%. These include components of efflux pump systems (mostly from RND family, i.e., MexAB-OprM, MexCD-OprJ, MexEF-OprN, MexGHI-OpmD, MexPQ-OpmE, MuxABC-OpmB, TriABC), multidrug transporter *emrE*, regulatory genes (*parRS, cpxR, cprRS, nfxB, nalC, nalD, mexT, mexS, mexZ, soxR, basS, rsmA*), membrane modification genes (*arnA, arnT*), and enzymatic resistance genes (*aph(3′)-IIb, fosA*).

Analysis of antimicrobial resistance determinants using ResFinder 4.5.0 identified several resistance-associated genes in both strains 304 and 448, including an aminoglycoside resistance gene (*aph(3′)-IIb*), beta-lactamase genes (*blaOXA-488* and *blaPAO*), a fosfomycin resistance gene (*fosA*), a chloramphenicol acetyltransferase gene (*catB7*), a ciprofloxacin resistance gene (*crpP*), and a tetracycline resistance gene (*tmexD2*).

Some of the detected genes have lower prevalence in *P. aeruginosa*: *blaOXA-488* (12.6%), *blaPDC-34* (2.8%), *adeF* (35.7%), *mexF* (73.3%), *mexZ* (77.9%). Additionally, *armR*, encoding a regulator of the MexXY-OprM efflux system, was found only in strain 448.

### Bacterial susceptibility to antibiotics

The findings highlight the presence of antibiotic-resistant *P. aeruginosa* strains in the cosmetics production line, posing a potential risk of contamination and infection. The MICs of ceftazidime (CAZ), gentamicin (GN), imipenem (IMP), and colistin were 2 to 32 µg mL^−1^, 2 to 64 µg mL^−1^, 2 to 32 µg mL^−1^ and 1 to 2 µg mL^−1^, respectively. Among the 5 tested strains, 3 were resistant to IMP: *P. aeruginosa* 2096, which was isolated from shower gel (MIC = 32 µg mL^−1^) and two *P. aeruginosa* strains (448 and 304), which were isolated from SLES1 and SLES2 (MIC = 16 µg mL^−1^). *P. aeruginosa* 2096 also showed resistance to CAZ (MIC = 32 µg mL^−1^). *P. aeruginosa* strains isolated from SLES1 and SLES2 were also resistant to GN (MIC = 32 µg mL^−1^ and 64 µg mL^−1^, respectively). The remaining two strains, the reference *P. aeruginosa* PAO1 and *P. aeruginosa* 1999 strains, which were isolated from a calendula extract, were susceptible to all the tested antibiotics. CL had the best antibacterial effect against all the tested *P. aeruginosa* rods. The results obtained indicate that antibiotic-resistant strains are present in the production lines of the cosmetics industry, highlighting the need for appropriate methods and means of sterilizing the production line to minimize the risk of cosmetic product contamination.

### Biofilm formation in the presence of disinfectants, cleaning and sanitizing agents

**Fig. 4:**
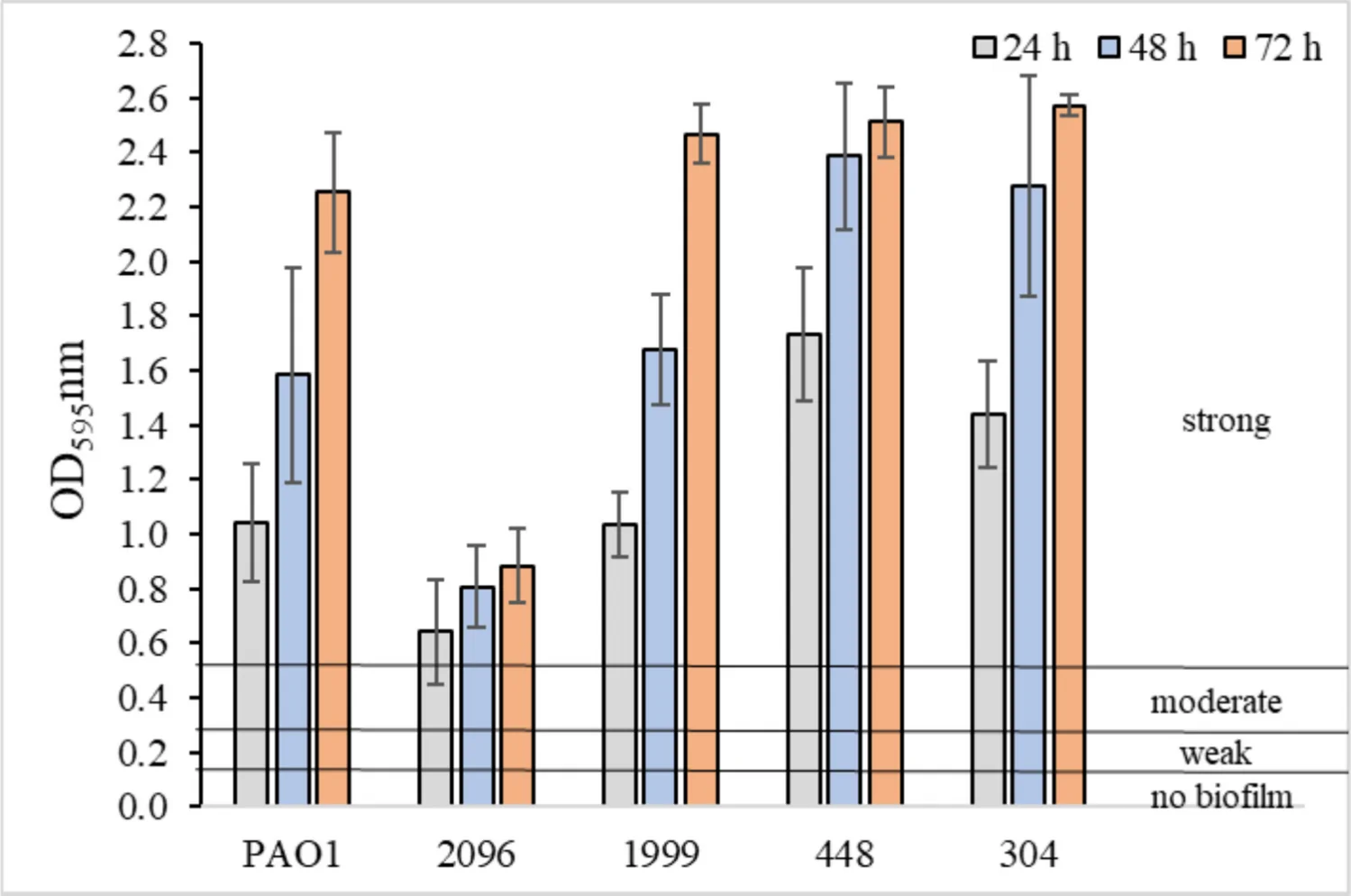
Biofilm formation by *P. aeruginosa* strains. Values represent means from three independent experiments

The obtained results indicate that all tested *P. aeruginosa* strains formed strong biofilms (OD > 0.560), regardless of the incubation time (Fig. 4).

**Fig. 5:**
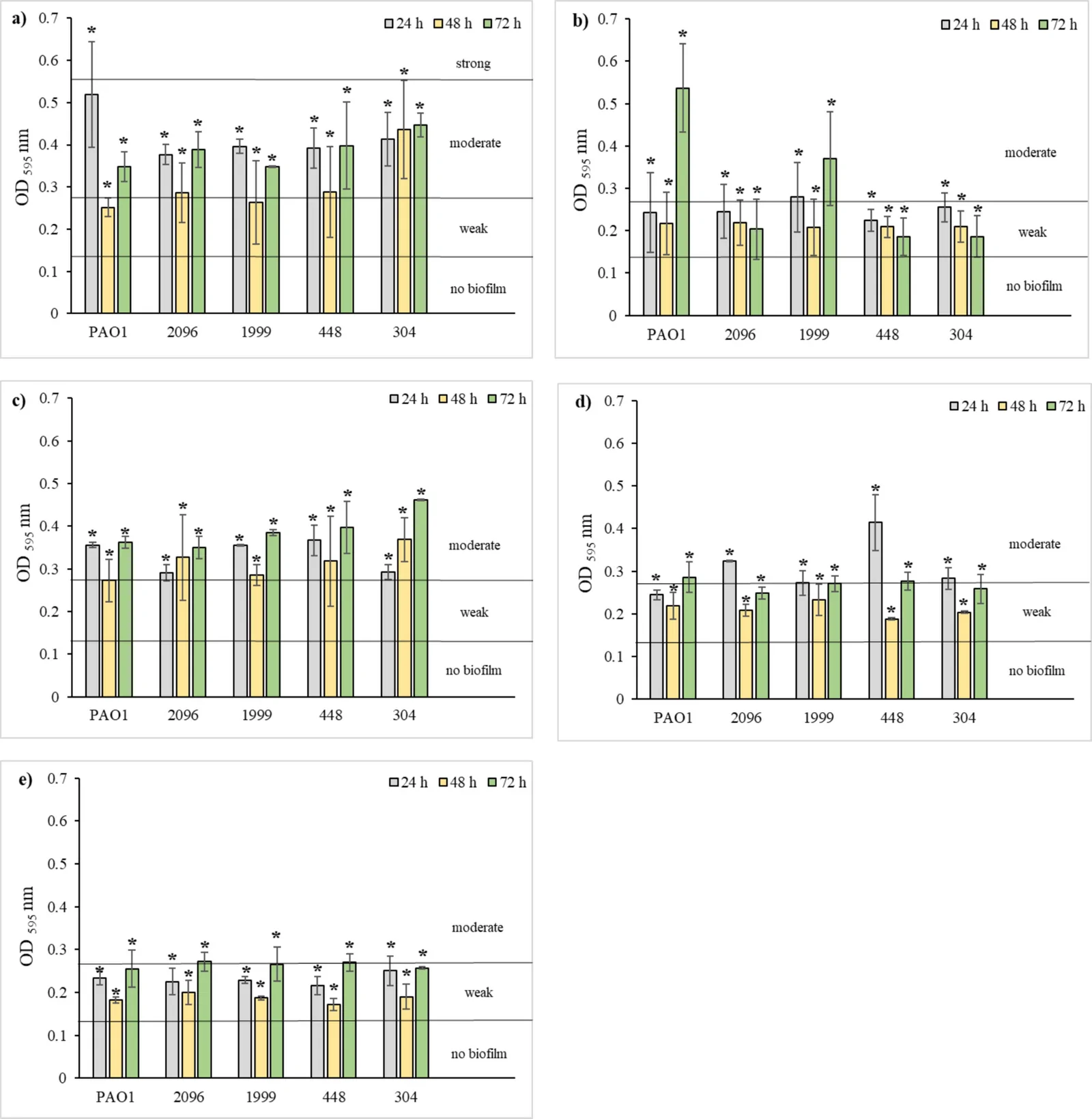
Biofilm formation by *P. aeruginosa* strains in the presence of cleaner agent (**a**), sanitizer agent (**b**), disinfectant 1 (**c**), disinfectant 2 (**d**), and disinfectant 3 (**e**). Values represent means from three independent experiments.* — the result was statistically significant compared to the control sample (*p* < 0.05)

As shown in Fig. 5, all tested disinfectants, cleaning and sanitizer agents exhibited anti-biofilm activity. They significantly reduced biofilm formation compared to the control samples (*p* < 0.05; Fig. 4). The most effective anti-biofilm activity was observed for hypochlorous acid (disinfectant 3), which resulted in weak biofilm formation (0.140 < OD ≤ 0.280), irrespective of the incubation time (Fig. 5e). Sanitizer agent and disinfectant 2 also demonstrated notable anti-biofilm effects (Fig. 5b and d). *P. aeruginosa* strains exposed to these agents typically produced weak biofilms (0.140 < OD ≤ 0.280), occasionally moderate ones (0.280 < OD ≤ 0.560) (*p* < 0.05). The weakest anti-biofilm activity was exhibited by the cleaning agent and disinfectant 1 (Fig. 5a and c). In their presence, biofilm formation was classified as moderate (0.280 < OD ≤ 0.560).

### Efficacy of disinfectant, cleaning and sanitizing agents on the metabolic activity of bacteria living in preformed biofilms

**Fig. 6:**
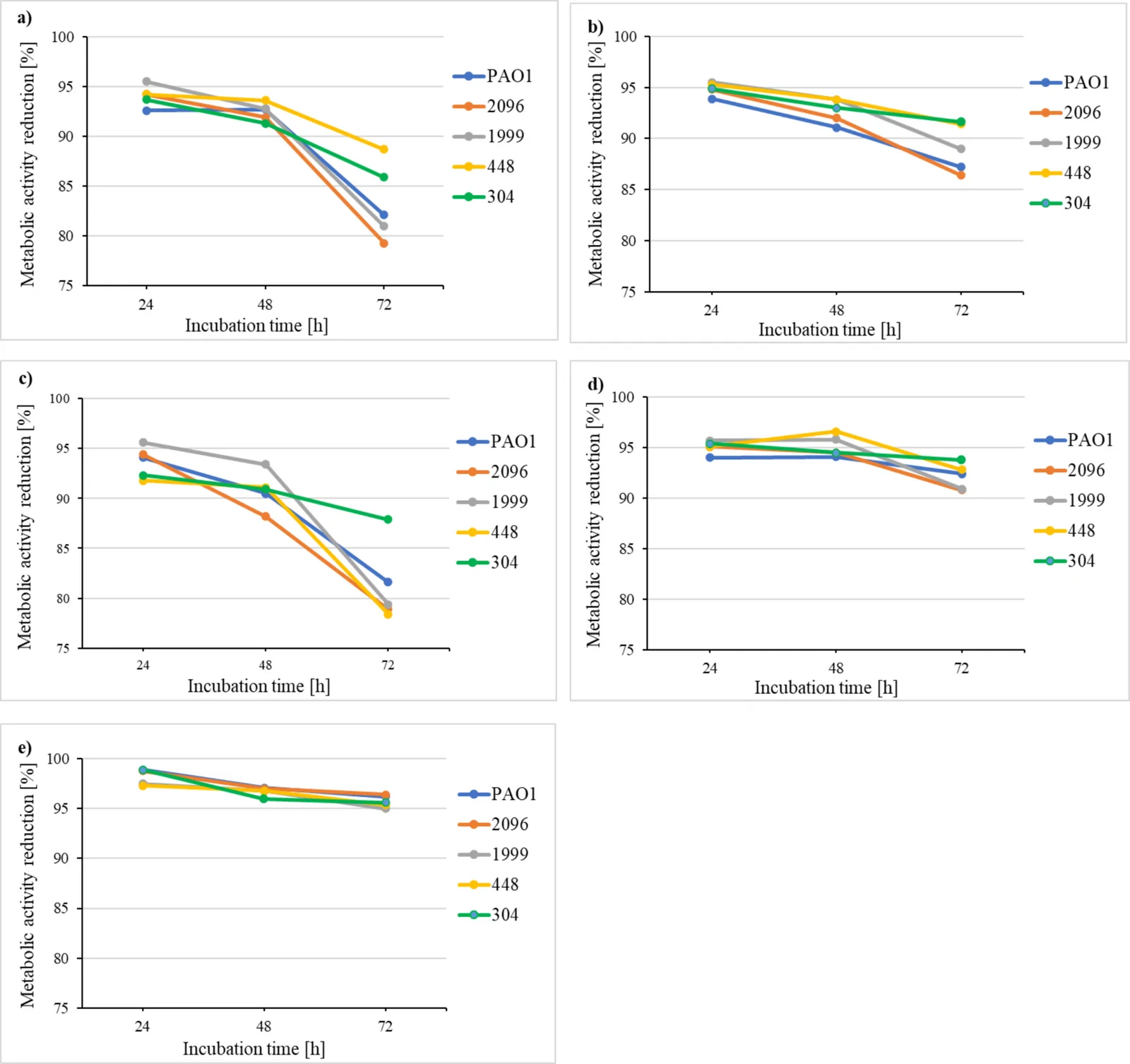
Metabolic activity reduction of *P. aeruginosa* rods living in biofilms by cleaner agent (**a**), sanitizer agent (**b**), disinfectant 1 (**c**), disinfectant 2 (**d**), and disinfectant 3 (**e**). Values represent means from three independent experiments

Figure 6 illustrates the reduction in the metabolic activity of biofilm-associated bacteria following treatment with a disinfectant, cleaning, or sanitizing agents.

The strongest antimicrobially-induced inhibition of metabolic activity was observed with disinfectant 3 (hypochlorous acid) (Fig. 6e). After 24 h of biofilm formation, the reduction in metabolic activity ranged from 97.3% to 98.9%. For 48-h and 72-h biofilms, this effect slightly diminished, with reductions ranging from 96.0% to 97.1% and from 95.0% to 96.4%, respectively.

Disinfectant 2 exhibited a slightly lower antimetabolic effect compared to disinfectant 3 (Fig. 6d). In the presence of disinfectant 2, metabolic activity in 24-, 48-, and 72-h biofilms was reduced by 94.0–95.7%, 94.1–96.6%, and 90.8–93.8%, respectively.

The cleaning agent, the sanitizer, and disinfectant 1 demonstrated comparatively weaker suppression of metabolic activity (Fig. 6a, b, and c). This effect declined progressively with increasing biofilm maturity. In 24-h biofilms, metabolic activity was reduced by 92.6% (cleaning agent, Fig. 6a) to 94.1% (disinfectant 1, Fig. 6c). In 48-h biofilms, the reduction ranged from 88.2% (disinfectant 1, Fig. 6c) to 93.6% (cleaning agent, Fig. 6a). The weakest inhibition was observed in 72-h biofilms, with reductions ranging from 78.4% (disinfectant 1, Fig. 6c) to 91.6% (sanitizer, Fig. 6b).

### Effects of disinfectants, cleaning and sanitizing agents on the metabolic activity of bacteria living in biofilms formed on silicone, Teflon, EPDM and acid-resistant steel surfaces

**Fig. 7:**
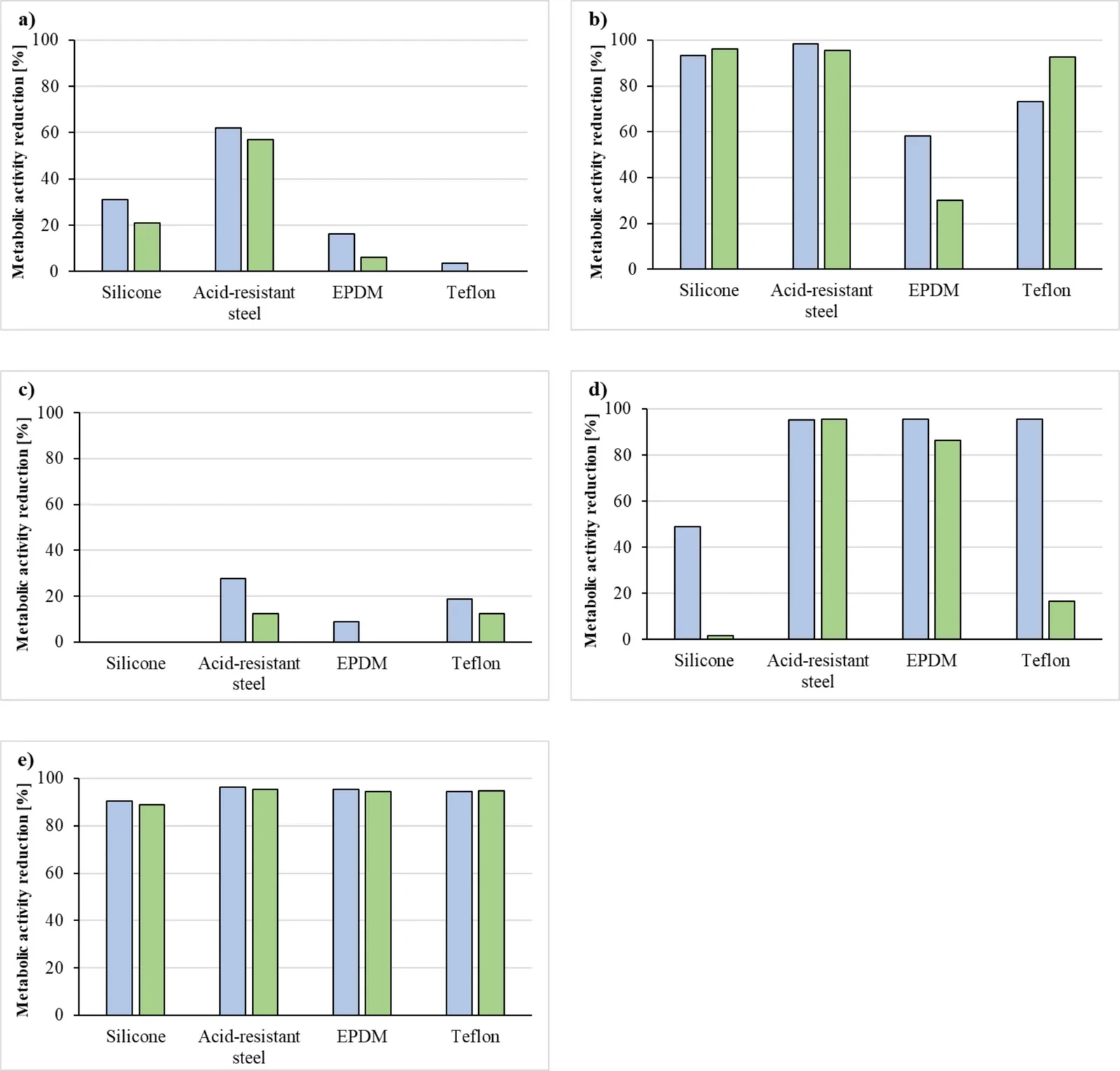
Metabolic activity reduction of *P. aeruginosa* strains SLES1 (blue bars) and SLES2 (green bars) by cleaner (**a**), sanitizer (**b**), disinfectant 1 (**c**), disinfectant 2 (**d**), and disinfectant 3 (**e**). Values represent means from three independent experiments

The results indicate that the degree of metabolic activity reduction in *P. aeruginosa* SLES1 and *P. aeruginosa* SLES2 living in 5-day biofilms depends on both the type of disinfectant, cleaning or sanitizing agent used and the type of surface (silicone, Teflon, EPDM or acid-resistant steel) (Fig. 7).

Disinfectant 3 caused the most effective metabolic activity reduction (Fig. 7e). The metabolic activity of *P. aeruginosa* SLES1 and *P. aeruginosa* SLES2 decreased from 90.5% (silicone gasket) to 96.2% (acid-resistant steel) and from 88.9% (silicone gasket) to 95.3% (acid-resistant steel), respectively. The least effective disinfectant against bacteria was disinfectant 1 (Fig. 7c). The metabolic activity reduction ranged from 0% (silicone gasket) to 27.6% (acid-resistant steel) against *P. aeruginosa* SLES1 and from 0% (silicone and EPDM gaskets) to 12.5% (acid-resistant steel) against *P. aeruginosa* SLES2.

The results shown in Fig. 7 indicate that the greatest reduction in metabolic activity in bacteria was observed in biofilms growing on acid-resistant steel regardless of the type of disinfectant. In contrast, the lowest reduction was observed for the silicone gaskets treated with cleaner agent, disinfectants 1 and 2 (Fig. 7a, c, and d), and the EPDM and Teflon gaskets treated with both the cleaner and disinfectant 1, respectively (Fig. 7a and c).

### Visualization of bacterial biofilms using SEM, EFM, and OCT

**Fig. 8:**
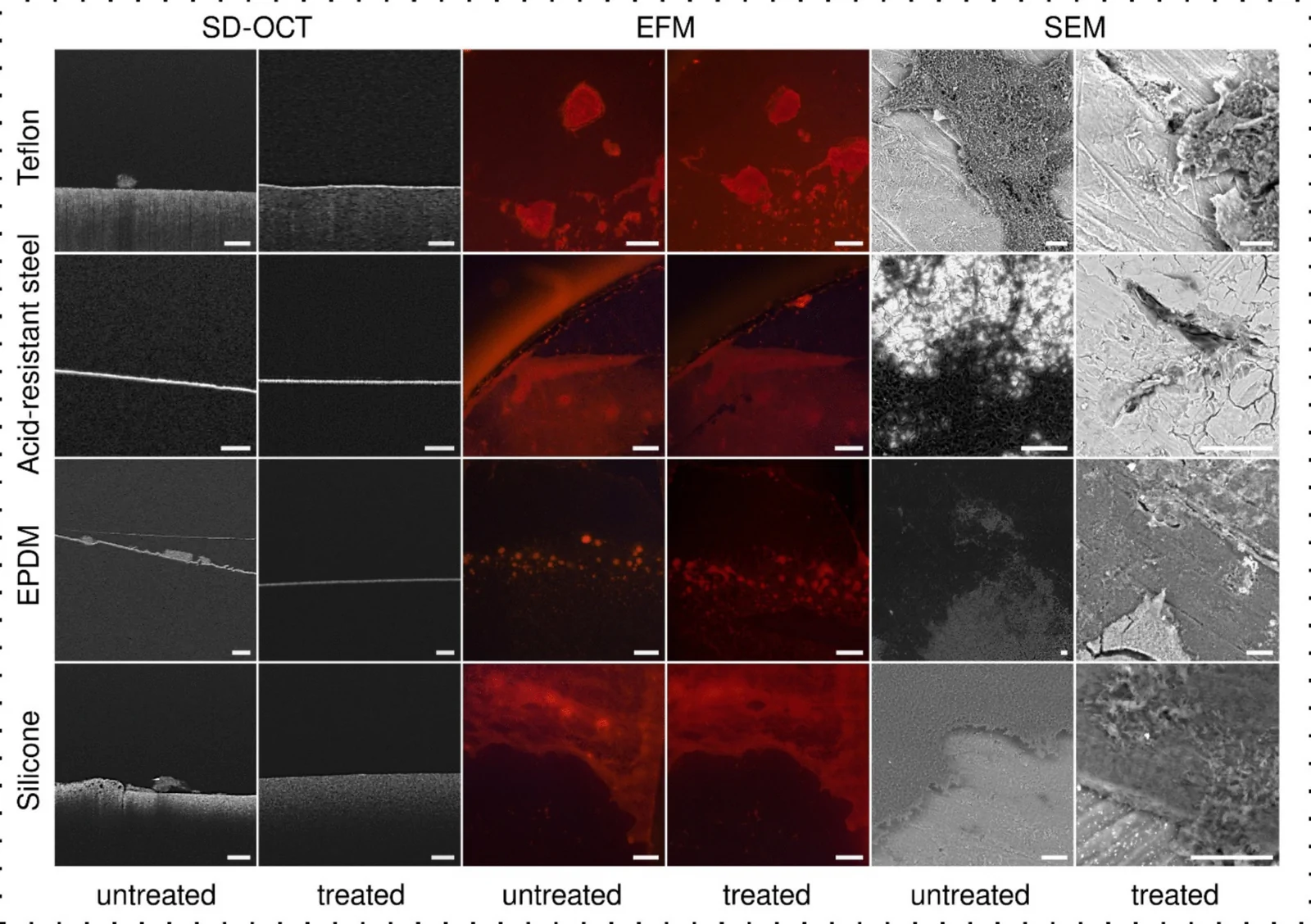
An assessment of biofilm formation on various surfaces and the effectiveness of its removal with hypochlorous acid. Spectral-Domain Optical Coherence Tomography (SD-OCT), Epifluorescence Microscopy (EFM), and Scanning Electron Microscopy (SEM) were employed to visualize biofilms on silicone, ethylene propylene diene monomer (EPDM), acid-resistant steel, and Teflon surfaces. Each row corresponds to a substrate with side-by-side comparisons of the biofilm before (untreated) and after (treated) hypochlorous acid treatment. Scale bar = 200 µm

SEM images revealed the detailed surface morphology of the biofilms and showed that the biofilm matrix degraded after hypochlorous acid treatment. This was consistent across all materials examined (Fig. 8). Despite this visible degradation, EFM results showed a biofilm layer on all materials after treatment, suggesting that some matrix components remained unaffected. However, the silicone surface showed a local decrease in fluorescence after treatment, confirming detachment of the biofilm. SD-OCT images provided further insight by revealing differences in biofilm biomass between untreated samples and samples treated with 100 ppm hypochlorous acid, indicating the removal of biofilms that were not firmly attached to Teflon, EPDM, or silicone. In contrast, the stainless steel surfaces contained no such detachable biofilm layer. Importantly, the residual biofilm layer detected by EFM could not be seen in the SD-OCT images, suggesting that these layers must be thinner than the axial resolution limit of SD-OCT (4.2 μm). In summary, visualization of biofilms on different surfaces before and after treatment with hypochlorous acid at a concentration of 100 ppm showed that the treatment removed most of the biofilm volume (SD-OCT) and degraded the biofilm matrix (SEM), but left a thin residual layer of organic matter that could bind the fluorescent stain (EFM).

### Use of hypochlorous acid (HClO) as a disinfectant in the water system of the production line

The results presented in the previous sections indicated that disinfectant 3 (HClO) was the most effective in limiting biofilm formation and the metabolic activity of viable bacterial cells. Therefore, HClO was selected for use in the water system of the production line. The results of the study indicated that the disinfection effect during the first two weeks of using HClO at a concentration of 2000 ppm lasted only two days (Table 3). In the following weeks (weeks 3–9), the disinfection effect lasted for three days. The CFU mL^−1^ value decreased significantly to 0.9 × 10^4^. During weeks 10 to 15, the disinfection effect was prolonged, lasting for four days. The level of contamination in the water flowing through the installation elements decreased from an uncountable value in 1 mL to a CFU count of 6.2 × 10^5^. Throughout weeks 1 to 15, the bacterial count in 1 mL was uncountable every Monday. From week 16 onwards, on the day HClO was dosed (Monday), the CFU count was 6.2 × 10⁶ (week 16), and over time, it decreased to 2.1 × 10^2^. In weeks 16 to 24, the disinfection effect lasted for seven days, from Monday to the following Monday, when HClO was again added to the water system. The hypochlorous acid used demonstrated high efficiency in removing resident microorganisms responsible for biofilm formation in the process water.

**Table 3:** Disinfecting effect of HClO in the water pumping system by 24 weeks

Week	CFU mL^−1^					Week	CFU mL^−1^				
	Before disinfection (Monday)	After disinfection [day]					Before disinfection (Monday)	After disinfection [day]			
		1	2	3	4			1	2	3	4
1	u.a	<0.1 × 10^1^	2.5 × 10^2^	u.a	u.a	13	u.a	<0.1 × 10^1^	1.8 × 10^2^	1.6 × 10^3^	2.8 × 10^5^
2	u.a	<0.1 × 10^1^	2.5 × 10^2^	u.a	u.a	14	u.a	<0.1 × 10^1^	3.5 × 10^2^	0.4 × 10^4^	1.4 × 10^5^
3	u.a	<0.1 × 10^1^	3.8 × 10^2^	6.0 × 10^4^	u.a	15	u.a	<0.1 × 10^1^	1.2 × 10^2^	1.2 × 10^4^	6.2 × 10^5^
4	u.a	<0.1 × 10^1^	3.5 × 10^2^	5.0 × 10^4^	u.a	16	6.2 × 10^6^	<0.1 × 10^1^	1.7 × 10^2^	6.8 × 10^3^	4.3 × 10^5^
5	u.a	<0.1 × 10^1^	2.8 × 10^2^	4.9 × 10^6^	u.a	17	4,8 × 10^6^	<0.1 × 10^1^	<0.1 × 10^1^	2.4 × 10^2^	4. × 10^3^
6	u.a	<0.1 × 10^1^	1.5 × 10^2^	4.8 × 10^5^	u.a	18	5,1 × 10^4^	<0.1 × 10^1^	<0.1 × 10^1^	5.4 × 10^2^	3.7 × 10^3^
7	u.a	<0.1 × 10^1^	1.8 × 10^2^	6.8 × 10^4^	u.a	19	4,2 × 10^5^	<0.1 × 10^1^	<0.1 × 10^1^	1.5 × 10^1^	4.8 × 10^3^
8	u.a	<0.1 × 10^1^	1.6 × 10^2^	1.8 × 10^4^	u.a	20	9.0 × 10^5^	<0.1 × 10^1^	<0.1 × 10^1^	2.7 × 10^1^	1.1 × 10^3^
9	u.a	< 0.1 × 10^1^	1.2 × 10^1^	0.9 × 10^4^	u.a	21	4,1 × 10^3^	<0.1 × 10^1^	<0.1 × 10^1^	3.1 × 10^1^	1.4 × 10^4^
10	u.a	<0.1 × 10^1^	4.8 × 10^1^	1.4 × 10^4^	6.5 × 10^5^	22	2,4 × 10^3^	<0.1 × 10^1^	<0.1 × 10^1^	<0.1 × 10^1^	1.2 × 10^1^
11	u.a	<0.1 × 10^1^	0.9 × 10^1^	2.6 × 10^3^	5.8 × 10^5^	23	3,6 × 10^2^	< 0.1 × 10^1^	<0.1 × 10^1^	<0.1 × 10^1^	0.3 × 10^1^
12	u.a	< 0.1 × 10^1^	2.8 × 10^2^	4.8 × 10^3^	4.9 × 10^5^	24	2.1 × 10^2^	< 0.1 × 10^1^	<0.1 × 10^1^	<0.1 × 10^1^	0.7 × 10^1^

The abbreviation u.a. means an uncountable amount

## Discussion

Microbiological contamination of cosmetics is an ongoing problem in the cosmetic industry. This phenomenon can occur at any stage of the manufacturing process because of contaminated raw materials or the production environment. Bacteria that are resistant to antibiotics and disinfectants are particularly dangerous for consumers’ health. The presence of microorganisms on production lines is a serious obstacle for manufacturers. The problem becomes more difficult to solve due to the ability of bacteria to produce biofilms that are resistant to cleaning agents and disinfectants (Rozman et al. 2021). For this reason, it is very important to indicate disinfectant compounds that have antibiofilm activity and can be implemented directly in production lines.

One of the pathogenic bacteria found in cosmetics is *P. aeruginosa*, which is inherently resistant to many antibiotics, capable of developing resistance to almost all available antibiotics, and capable of forming a biofilm that impedes the penetration of antibiotics (Akhand et al. 2023; Fernández-Billón et al. 2023). The formation of biofilms is currently the most serious problem in the cosmetics industry due to difficulties in their removal; therefore, cosmetic producers must constantly monitor the microbiological purity of the production process. The formation of biofilms is a relatively quick process, but its removal is very problematic because the formation of this structure significantly reduces the effectiveness of disinfectants. The physicochemical barrier constituted by extracellular polymeric substances, which are part of the matrix surrounding bacterial cells in biofilms, hinders the penetration of substances^.^ (Kostakioti et al. 2013; Muhammad et al. 2020).

Cleaning and disinfecting agents containing peroxygen compounds such as hydrogen peroxide, peracetic acid, or sodium hypochlorite; phosphoric acid; sodium hydroxide; and alcohols such as ethanol or 2-propanol are commonly used to remove bacterial biofilms from cosmetic manufacturing sites (Ludensky 2003). However, the increasing resistance of biofilms to antimicrobial agents has necessitated the search for more effective compounds for biofilm removal (Giaouris and Simões 2018).

Our studies determined the effects of cleaner containing sodium alkylbenzenesulphonate, tetrasodium ethylene diamine, and tetraacetate; sanitizer containing hydrogen peroxide, acetic acid, and peracetic acid; disinfectant 1 containing N-alkyl dimethyl benzyl and ammonium chloride; disinfectant 2 containing hydrogen peroxide; disinfectant 3 containing hypochlorous acid on biofilm formation and bacterial metabolic activity in biofilms. The obtained results indicated that the most effective antibiofilm effect and anti-metabolic activity were demonstrated by hypochlorous acid.

Lineback et al. (2018) demonstrated that hydrogen peroxide and sodium hypochlorite-based disinfectants were more effective against *P. aeruginosa* and *S. aureus* biofilms than were quaternary ammonium-based disinfectants (Lineback et al. 2018). Hydrogen peroxide and sodium hypochlorite-based disinfectants destroy both the biofilm matrix and the bacterial cells within it, making them good antibiofilm agents (De Queiroz and Day 2007). Presterl et al. (2007) demonstrated that hydrogen peroxide was effective in eliminating biofilms formed by *Staphylococcus epidermidis* (Presterl et al. 2007). Rushdy and Othman (2011) tested the antibiofilm activity of 30% H_2_O_2_ (hydrogen peroxide), 10% NaOH (sodium hydroxide), 70% C_2_H_5_OH (ethanol), 4–6% NaOCl (sodium hypochlorite), C_3_H_7_OH (isopropyl alcohol), mixtures of H_2_O_2_ and NaOCl, H_2_O_2_ and C_2_H_5_OH, H_2_O_2_ and C_3_H_7_OH against Gram-positive and Gram-negative bacteria growing in biofilms on stainless steel slides (Rushdy and Othman 2011). Hydrogen peroxide was found to be the most effective disinfectant for all bacteria tested. Oxidizing agents, which include hydrogen peroxide and chlorine-containing compounds, are usually low molecular weight compounds that easily penetrate bacterial envelopes (cell walls and membranes) and are then able to react with internal cellular components, leading to apoptotic and necrotic cell death (Finnegan et al. 2010).

Different results were obtained by Elkins et al. (1999), who reported a lack of antibiofilm effects of hydrogen peroxide on biofilms formed by *P. aeruginosa* (Elkins et al. 1999). In these biofilms, increased catalase activity was detected, which may be related to the protective mechanism of the bacteria and the ineffectiveness of this disinfectant. Stewart et al. (2000) also confirmed that catalase protects aggregated bacteria by preventing full penetration of hydrogen peroxide into the biofilm (Stewart et al. 2000). Genomic studies carried out by Weiser et al. (2019) on industrial strains of *P. aeruginosa* have shown that they have significantly larger genomes than reference *P. aeruginosa* strains do and that they also carry megaplasmids, which may be associated with increased resistance to preservatives and disinfectants and therefore better survival in stressful environments (Weiser et al. 2019).

Peracetic acid is particularly effective because it penetrates the biofilm’s protective matrix and generates reactive oxygen species that cause significant damage to bacterial cells. Chino et al. (2017) reported that peracetic acid can effectively damage *P. aeruginosa* and *S. aureus* biofilms within minutes, making it a potent biocide for biofilm eradication (Chino et al. 2017). However, Królasik et al. (2010) reported that the reduction in the number of bacteria in biofilms under the influence of a disinfectant containing hydrogen peroxide, peracetic acid, and octanoic acid depends on its concentration and duration of action (Królasik et al. 2010). A 0.5% disinfectant for 10 min was ineffective against biofilms. Disinfection effectiveness was achieved after a twofold increase in agent concentration and extension of the action time to 30 min.

Our studies have shown that disinfectant 1, which contains n-alkyl dimethyl benzyl ammonium chloride, has the weakest antibiofilm activity. This compound is a quaternary ammonium compound. Lineback et al. (2018) reported that quaternary ammonium compounds are characterized by weak antimicrobial activity against *P. aeruginosa* biofilms (Lineback et al. 2018).

The mechanisms of resistance of *P. aeruginosa* to disinfectants based on quaternary ammonium compounds have been described in the literature for many years (Guerin‐Mechin et al. 1999; Jones et al. 1989). The results of a study by Tabata et al. (2003) suggested that the adaptation of *P. aeruginosa* to this group of biocides was readily achieved by continuous contact with them. Changes in membrane fatty acid composition (Guerin‐Mechin et al. 1999) and increased expression of the outer membrane protein OprR (Tabata et al. 2003) are considered the main causes of resistance. This was also confirmed by our results, which revealed increased resistance of *P. aeruginosa* strains to a disinfectant based on quaternary ammonium compounds and to a disinfectant based on sodium dodecylbenzenesulfonate and tetrasodium EDTA. The strains isolated from SLES were characterised on a genetic level, revealing a rather common set of antimicrobial resistance genes, where most of them are present in more than 95% of *P. aeruginosa* genomes. Considering resistance to QACs, the strains have at least seven complete RND-family efflux pump systems. Among these, the MexAB-OprM system is constitutively expressed and serves as the principal mechanism for intrinsic resistance to benzalkonium chloride and other QACs (Amsalu et al. 2020). Beyond RND pumps, both strains have additional efflux mechanisms: *emrE* gene that encodes a small multidrug resistance family transporter that effluxes QACs (Spreacker et al. 2024), and *pmpM* gene that encodes a MATE-family efflux pump with confirmed activity against benzalkonium chloride (He et al. 2004). Some less prevalent genes were detected in the SLES strains, such as OXA-488 and PDC-34 beta-lactamases, although these genes are not particularly important for disinfectant resistance.

Although industrial disinfectants have antimicrobial activity, this activity may be limited by the biofilm structure. To increase the effectiveness of disinfectants, the biofilm should first be removed mechanically in the washing process (Guerin‐Mechin et al. 1999; Exner et al. 1987). Exner et al. (1987) studied the effects of disinfectants on biofilms formed in water supply systems (Exner et al. 1987). The authors reported that aldehyde- and peracetic acid-based agents caused the removal of microorganisms in water supply systems but did not completely remove biofilms. On the other hand, the hydrogen peroxide-based agent showed good antimicrobial efficacy and biofilm reduction. However, the highest efficacy of disinfectants was achieved when decontamination procedures were previously performed with cleaning agents that mechanically removed the adhering biofilm.

Our results also indicate that all the agents we used, with the exception of disinfectant 1, which contains n-alkyl dimethyl benzyl ammonium chloride, reduced the metabolic activity of bacteria living in the biofilm consortia. The effects of disinfectants on the metabolic activity of bacteria within biofilms were measured via the triphenyl tetrazolium chloride (TTC) method. TTC is a colorimetric assay that assesses metabolic activity by detecting the reduction of TTC to formazan, a process that occurs in active, living cells. The amount of formazan produced is proportional to the number of actively metabolizing cells, which allows the assessment of the viability and metabolic activity of bacteria in the biofilm (Haney et al. 2021). Disinfectants can significantly reduce the metabolic activity of bacterial cells in biofilms without necessarily reducing the biofilm mass. This phenomenon occurs because, while disinfectants can impair the metabolic functions of bacteria, the biofilm's extracellular polymeric substance (EPS) matrix, which provides structural integrity, often remains largely unaffected. Therefore, the biofilm still exists, but the bacteria within it are metabolically less active. Mougin et al. (2024) reported that benzalkonium chloride reduces bacterial metabolic activity without corresponding reductions in biofilm biomass (Mougin et al. 2024). Therefore, both biofilm mass and metabolic activity should be taken into account when assessing the effectiveness of disinfectants.

Our in vitro studies revealed that the metabolic activity of *P. aeruginosa* SLES1 and SLES2 present in biofilms formed on all three gaskets and stainless steel was significantly reduced only in the presence of hypochlorous acid (disinfectant 3). Hypochlorous acid is a well-established, concentration-dependent, nontoxic, easy-to-use, and safe alternative to other disinfectants based on chlorine compounds. The European Chemicals Agency (ECHA) has recognized hypochlorous acid as a biocidal product suitable for use in 5 ranges: human hygiene, surface disinfection, veterinary hygiene, food, and drinking water. The inert hypochlorous acid molecule possesses good penetrating properties for bacterial cell walls and pathogen cell membranes (da Cruz Nizer et al. 2020). Romanowski et al. (2018) reported that a hypochlorous acid solution (0.01%) was able to kill gram-positive *Staphylococcus* bacteria and gram-negative *P. aeruginosa* bacteria living in biofilms, but did not disrupt the biofilm structure (Romanowski et al. 2018). Hypochlorous acid effectively reduced the number of *S. aureus* bacteria and disrupted the polysaccharide and protein matrix in the biofilm model. Approximately 70% of biofilm polysaccharides and >90% of biofilm protein were removed (Robson 2014). Similar data were noted in the *P. aeruginosa* biofilm model (Sampson 2008).

For years, hypochlorous acid has been used to remove bacterial biofilms. Previous technologies produced hypochlorous acid with a large amount of free chlorite and sodium hypochlorite—a substance with strong potential to irritate the skin of humans. Notably, in our research, we used hypochlorous acid produced according to a new technology in the process of sodium chloride electrolysis, where only trace residues of free chlorine and sodium hypochlorite are produced. Owing to this new technology, hypochlorous acid can be used for water sanitization, water circulation, and all surfaces in the cosmetics industry. This technology is also used in cosmetic production plants from which the *P. aeruginosa* strains we tested were obtained.

In many technological processes, microorganisms are not a problem as long as they remain in planktonic form. Disinfection might be easier if the attachment of microbes is limited. Therefore, the type of surface material is one of the key factors responsible for the ability of bacteria to settle on it and form a biofilm (Meyer 2003). The impact of surface materials such as silicon, Teflon, and stainless steel on bacterial biofilm formation varies due to their distinct physical and chemical properties. When modified, silicon surfaces can sometimes reduce bacterial adhesion. For example, adding a nanoscale silicon dioxide coating to stainless steel can lead to lower bacterial attachment but does not completely prevent biofilm formation by *Listeria* monocytogenes (Hillig et al. 2024). Teflon, known for its nonstick properties, tends to resist biofilm formation better than many other materials do. However, their effectiveness varies depending on the environmental conditions and bacterial species. When combined with stainless steel, it can further reduce the bacterial adhesion of *E. coli* and *Salmonella* Typhimurium (Ban et al. 2020). Stainless steel is widely used in industry but is susceptible to biofilm formation. The microtopography of stainless steel examined by SEM and AFM reveals cracks and crevices that may increase the surface area for the attachment of bacterial cells and allow them to be protected from cleaning/disinfection agents (Palmer et al. 2007). Fatemi and Frank (1999) demonstrated that biofilms of mixed cultures of *Pseudomonas* spp. and *Listeria monocytogenes* formed on stainless steel were inactivated by peracetic acid and peroctanoic acid-based disinfectants (Fatemi and Frank 1999). Rossoni and Gaylarde (2000) reported that sodium hypochlorite was more effective than peracetic acid in killing or removing *E. coli*, *Pseudomonas fluorescens*, and *S. aureus* adhering to stainless steel (Rossoni and Gaylarde 2000).

Determining the drug sensitivity/resistance of the tested *P. aeruginosa* strains was an important epidemiological aspect of our studies. Drug-resistant pathogens are particularly dangerous to human health and life. The presence of such microorganisms in water system installations at a production plant creates a risk of their entry into production wastewater and from there into the environment. *P. aeruginosa* rods can exhibit resistance to many antibiotics (Martins et al. 2018; Shaqra et al. 2014). The three *P. aeruginosa* strains used in our studies were resistant to one or more of the antibiotics used, such as ceftazidime, gentamicin, and imipenem. Antibiotic resistance in bacteria found in cosmetic products is an emerging concern. Studies have shown that preservatives such as triclosan and parabens, which are commonly used in cosmetics, can contribute to the development of bacterial resistance (Caioni et al. 2023). These substances can cause bacteria to develop cross-resistance to both preservatives and antibiotics, such as β-lactams and quinolones. For example, strains such as *S. aureus*, *P. aeruginosa*, and *Enterobacter gergoviae* found in contaminated cosmetics have shown increasing antibiotic resistance (Rybczyńska-Tkaczyk et al. 2023). Cases of resistance acquisition by antibiotic-susceptible *P. aeruginosa* strains in the presence of disinfectants have also been reported in the literature (Martins et al. 2018; Noaman et al. 2023). An analysis of the results of our studies revealed no correlation between the resistance of the tested *P. aeruginosa* strains living in biofilm consortia to disinfectants and their resistance to antibiotics. All the tested strains were resistant to the cleaning agent and disinfectant 1, and only three of them were characterized by resistance to antibiotics.

## Conclusion

In the cosmetics industry, microbiological contamination of raw materials and technological equipment poses a significant problem, potentially leading to the contamination of cosmetic products with pathogenic microorganisms. In this work, hypochlorous acid proved to be the most effective in inhibiting biofilm formation and reducing the metabolic activity of *P. aeruginosa* strains. It significantly suppressed the activity of biofilm-forming bacteria on all tested surfaces, including silicone, acid-resistant steel, EPDM, and Teflon. The greatest reduction in bacterial metabolic activity was observed in biofilms formed on acid-resistant steel, regardless of the type of disinfectant used. The application of hypochlorous acid in the water system of a cosmetics manufacturing facility confirmed its high efficacy in eliminating resident microorganisms responsible for biofilm formation.

## Data Availability

All data generated or analyzed during this study are included in this article.
